# Mitral regurgitation and all-cause mortality in acute heart failure: a meta-analysis

**DOI:** 10.1093/eschf/xvag200

**Published:** 2026-08-04

**Authors:** Max Berrill, Meric Bengisu, Konstantina Kipourou, Gie Ken Dror, Paula Coles, Isaac John, Pankaj Sharma, Aigul Baltabaeva

**Affiliations:** Department of Research & Development, St Peter’s Hospital, Chertsey KT16 0PZ, UK; Department of Echocardiography, Harefield Hospital, Harefield UB9 6JH, UK; Department of Echocardiography, Harefield Hospital, Harefield UB9 6JH, UK; Department of Echocardiography, Harefield Hospital, Harefield UB9 6JH, UK; Institute of Cardiovascular Research, Holloway University, London, UK; Bart’s Health Knowledge and Library Services, St Bartholomew’s Hospital, London, UK; Department of Research & Development, St Peter’s Hospital, Chertsey KT16 0PZ, UK; Institute of Cardiovascular Research, Holloway University, London, UK; Department of Research & Development, St Peter’s Hospital, Chertsey KT16 0PZ, UK; Institute of Cardiovascular Research, Holloway University, London, UK; Department of Research & Development, St Peter’s Hospital, Chertsey KT16 0PZ, UK; Department of Echocardiography, Harefield Hospital, Harefield UB9 6JH, UK

**Keywords:** MR, Mitral regurgitation, Heart failure, Acute heart failure, Systematic review, Meta-analysis

## Abstract

**Introduction:**

Despite previous studies investigating the impact of mitral regurgitation (MR) in acute heart failure (AHF) there are inconsistencies in trial data, and no meta-analyses to date. Our aim was to explore if MR is associated with mortality in AHF.

**Methods and Results:**

We performed a meta-analysis of AHF patients with MR to investigate any association with all-cause mortality. We identified 27 studies that met our inclusion criteria, encompassing 73 363 patients (age 72.9 ± 4.2years; females 44.4%) with an average follow-up of 1.9 years. Moderate/severe MR was associated with an unadjusted 47% increased mortality risk compared with no/mild MR (odds ratio [OR] 1.47 [95% confidence interval {CI} 1.30–1.66, *P* < .001]). This excess risk of death persisted among studies reporting adjusted data (OR 1.21 [95% CI 1.01–1.45, *P* = .038]). Compared with those with no MR, MR severity was associated with increasing risk of death: mild MR OR 1.22 [95% CI .98–1.53, *P* = .079], moderate MR OR 1.48 [95% CI 1.1–2.01, *P* = .011], and severe MR OR 1.60 [95% CI 1.13–2.27, *P* = .008]. We observed excess mortality in moderate/severe MR compared with no/mild MR among individuals with a left ventricular ejection fraction (LVEF) < 50% (OR 1.26 [95% CI 1.18–1.35, *P* < .001]) and LVEF 40–50% (OR 2.00 [95% CI 1.59–2.50, *P* < .001]) but not LVEF >50% (OR 1.16 [95% CI .91–1.47, *P* = .23]. Neither meta-regression nor prespecified sensitivity analyses affected MR’s association with the primary outcome. A substantial degree of heterogeneity was observed across the included studies.

**Conclusions:**

We observed that MR during AHF is associated with increased mortality. Although causality cannot be inferred due to the observational design and high heterogeneity of the included studies, MR may represent a phenotype amenable to targeted therapies during decompensated heart failure.

## Graphical Abstract

**Graphical Abstract:**
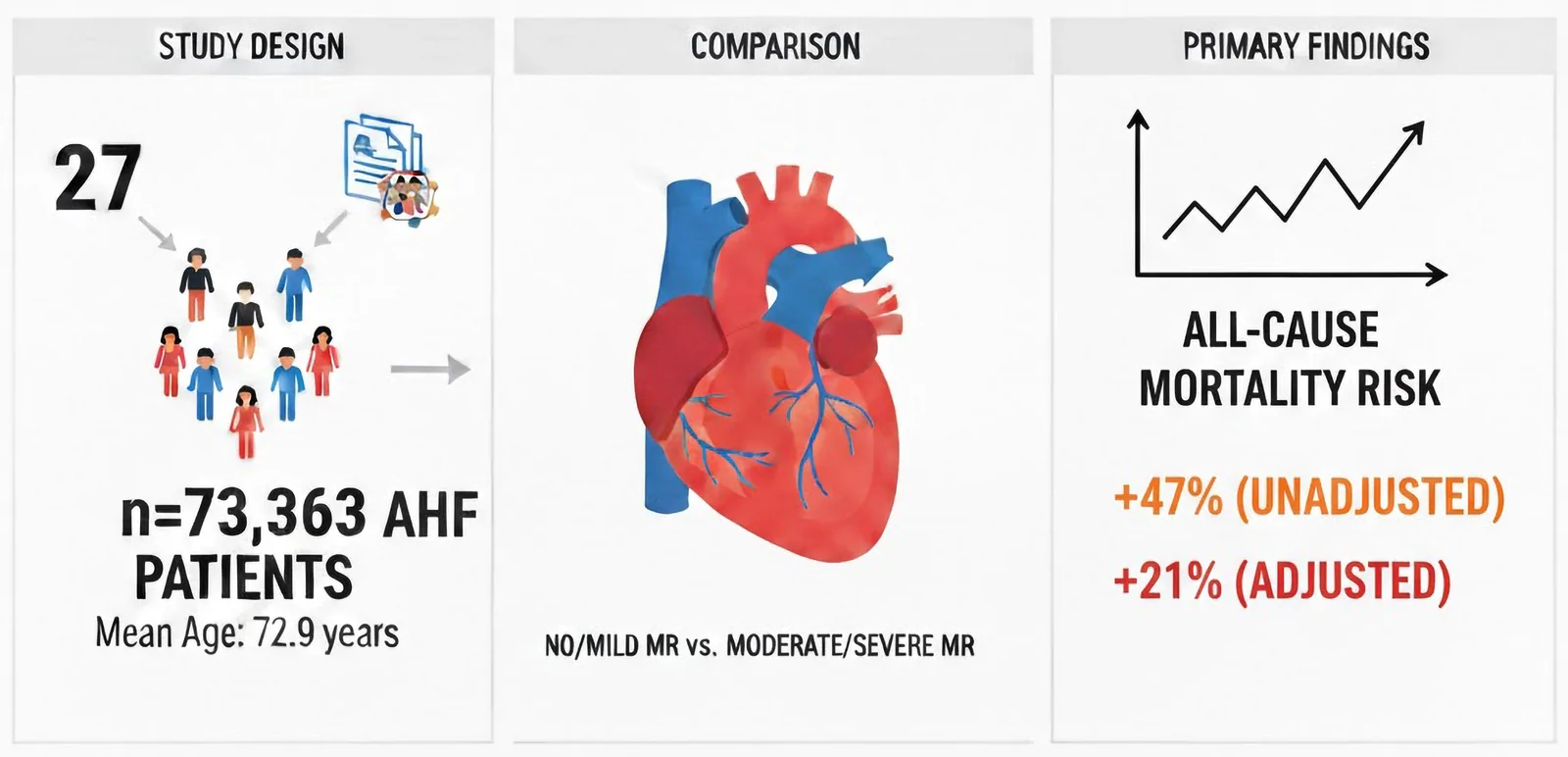

## Introduction

Acute heart failure (AHF) is a leading cause of hospitalization worldwide^1^ with mitral regurgitation (MR) the most common valvular disorder identified during in-patient echocardiography among European patients.^2^

Despite this prevalence, an association between MR and outcome in AHF is poorly understood. Previous studies suggest an association between even mild MR and mortality^3^ but inconsistencies in trial data are observed when investigating MR’s prognostic association among those with either a reduced or a preserved left ventricular ejection fraction (LVEF)^4^ and when an effect of MR on outcome is adjusted for other risk factors.^5,6^

We undertook a meta-analysis to clarify whether MR is associated with mortality in AHF.

## Methods

### Search strategy

This study was pre-registered (CRD42024612424) and designed to meet the recommendations of both the PROSPERO systematic review registration and Meta-analyses in Observational Studies in Epidemiology requirements. Our hypothesis prior to registration and data collection was that MR would be associated with mortality. We assessed Medline and EMBASE via the OVID platform and Cochrane Library and PubMed online databases were searched for articles (including abstracts and conference proceedings) published up to January 2025. The search was designed and tested in Medline and peer reviewed by a medical specialist librarian. We screened only articles published in English.

We included studies which met the inclusion criteria and resolved any disagreements by consensus. Two authors with expertise in echocardiography independently performed screening and assessed eligibility. We undertook manual searches of references and citations of the initial articles which met our inclusion criteria. Apart from automatic deduplication by referencing software, all exclusions were by hand. We did not contact authors directly.

### Inclusion criteria

We included studies in which patients had an unplanned presentation with worsening signs and symptoms of heart failure (HF) requiring initiation or intensification of HF treatment. Mitral regurgitation needed to be assessed using echocardiography during their decompensation, this could be either transthoracic or transoesophageal. All-cause mortality events or ORs (odds ratios) needed to be provided, as well as the degree of MR.

We excluded studies of transcatheter or surgical mitral valve repair or replacement, patients receiving mechanical circulatory support, paediatric patients, studies of acute rheumatic fever, acute Takotsubo cardiomyopathy, studies of acute, severe MR due to acute coronary syndrome with papillary muscle rupture, MR related to infective endocarditis, and MR in the context of complex congenital heart disease.

### Data extraction

Two authors independently extracted study characteristics, demographic, comorbidity, and echocardiography data, ORs and the corresponding confidence intervals (CIs) for each study. To evaluate the impact of confounding, we extracted adjusted ORs and, where studies performed multiple analyses, we selected the model with maximal adjustment. We only extracted adjusted data for the primary analysis. When providing all-cause mortality with differing lengths of follow-up, we included the longest duration. Among studies which assessed MR serially, we preferentially used the admission echocardiogram, except if later data were used in a multivariate analysis.

### End points and definitions

End point criteria was all-cause mortality with severity of MR. The primary analysis was comparison between no/mild MR and moderate/severe MR. Secondary analyses included the incidence of all-cause mortality and severity of MR and among those an LVEF <50%, LVEF >50%, and an LVEF 40%–50%. Acute heart failure was defined as individuals presenting with worsening HF signs or symptoms such that individuals sought unplanned medical attention with a corresponding intensification of treatment. Mitral regurgitation had to be assessed by transthoracic echocardiography during the episode of decompensation. Mitral regurgitation evaluation was defined as qualitative if limited to visualization of the colour flow jet area, semi-quantitative if assessed with vena contract width, proximal isovelocity surface area, pulmonary venous flow assessment, or the proportion of MR jet occupying left atrial area and quantitative if the effective regurgitant orifice or regurgitant volume was calculated.

### Study quality evaluation

We evaluated risk of bias for each study using the Newcastle–Ottawa Scale (NOS), a tool previously evaluated for inter-rater reliability. Studies were evaluated and scored on three domains (selection, comparability, and outcome) with total score of 9 available (Supplementary Appendix). The overall risk of bias for the study was considered high if any individual domain was graded as high or if the total score was ≤4, moderate risk if 5–7, and low risk if 8–9. We considered studies of specific LVEF phenotypes to still be representative of AHF in general and considered the most important comparability factor to control for to be age.

### Statistical analysis

Odds ratios were used on the original measurement scale to determine the magnitude of the impact of MR on mortality. Pooled estimates of outcome were obtained via the generalized inverse variance method using a fix effects model on the original scale of measurement or the DerSimonian and Laird method using a random effects model in the case of significant heterogeneity (I^2^ > 30%). The I^2^ statistic and Cochran’s Q test assessed heterogeneity of trial results. We performed sensitivity analyses including evaluation of adjusted data to allow a comparison between adjusted vs unadjusted associations to assess any degree of confounding. We performed further analyses removing trials deemed at high risk of bias (as determined by the NOS rating) and another analysis removing trials at high or moderate risk of bias. We also performed leave-one-out meta-analyses with sequential removal of each study in turn, to identify if any single study removal impacts on the overall findings. Potential publication bias was estimated both visually and by a linear regression approach. Meta-regression analyses were performed incorporating prespecified covariates deemed by the authors to have likelihood of impacting the primary outcome. This included age, gender, a history of hypertension, diabetes mellitus, or atrial fibrillation. Additionally, we assessed length of follow-up and its association with the primary outcome. A *P* < .05 was considered statistically significant. Analyses, table, and graphic generation were performed using R version 4.5.0 (R Foundation for Statistical Computing, Vienna, Austria).

## Results

### Study summary

We identified 5236 articles, of which 27 (73 363 patients, average follow-up of 1.9 years) met the inclusion criteria (*Figure 1*). Summary characteristics of study population are displayed in *Table 1*.

**Figure 1:**
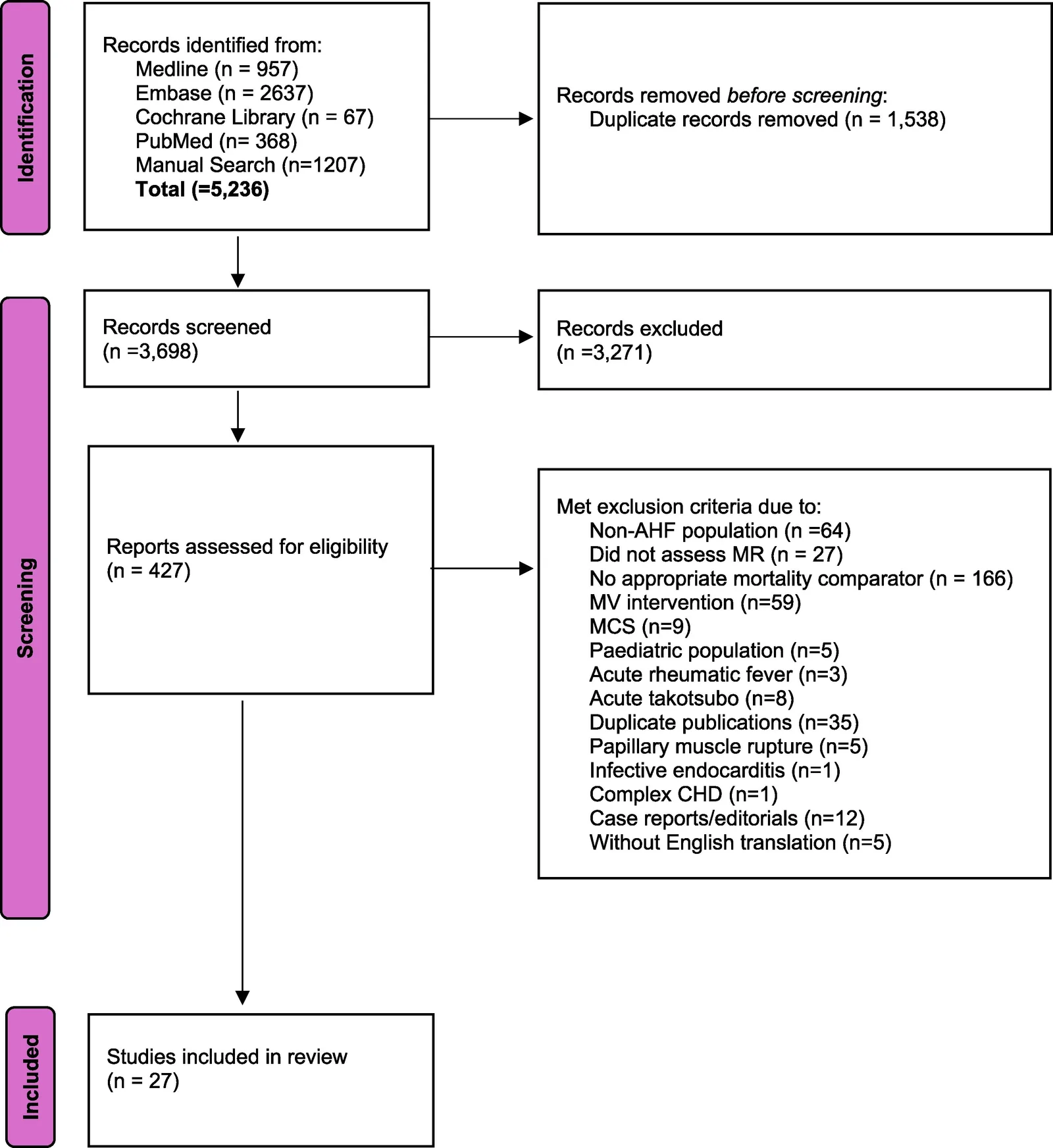
PRISMA flow diagram

**Table 1:** The baseline data of study population

Variable	Total population (SD)	No/mild MR (SD)	Moderate/severe MR (SD)	*P*-value
Age (years)	72.9 (4.2)	68.5(11.4)	73.6 (2.9)	<.001
Female gender (%)	44.4 (9.6)	43.7 (9.6)	46.6 (9.8)	<.001
HTN (%)	77.6 (11.7)	79.6 (10.6)	75.4 (13)	<.001
DM (%)	41.6 (6.8)	44.2 (8.5)	37.8 (4.9)	<.001
CAD (%)	54.1 (8.1)	53.2 (7.2)	56.4 (9.6)	<.001
CVA (%)	16.6 (2.9)	16.8 (2.6)	16.2 (3.7)	<.001
MI (%)	27.1 (6.6)	26.1 (7.1)	29.2 (6.8)	<.001
CKD (%)	41.8(17.1)	42.6 (17.2)	41.5 (17.2)	<.001
AF (%)	39.6 (7.5)	36.8 (7.1)	44.9 (9.6)	<.001
COPD (%)	22 (8.2)	22.5 (8.8)	20.6 (7.6)	<.001
LVEF (%)	41.1 (6.5)	41.7 (7)	35.9 (5.2)	<.001
LVEDD (cm)	53.7 (3.5)	52.8 (4.2)	57.4 (4.0)	<.001
LA (cm)	4.4 (.2)	4.4 (.2)	4.8 (.1)	<.001
SPAP (mm Hg)	47.3 (3.1)	43.1 (3.8)	49.8 (4.2)	<.001

MR, mitral regurgitation; HTN, hypertension; DM, diabetes mellitus; CAD, coronary artery disease; CVA, cerebrovascular accident; MI, myocardial infarction; CKD, chronic kidney disease; AF, atrial fibrillation; COPD, chronic obstructive pulmonary disease; LVEF, left ventricular ejection fraction; LVEDD, LV end-diastolic diameter; LA, left atrium; SPAP, systolic pulmonary artery pressure.

### Primary analysis

Twenty-two^4–24^ (*n* = 51 922) studies had extractable data for the primary analysis. Among these studies moderate/severe MR was associated with a 47% increased unadjusted risk of all-cause mortality compared with no/mild MR (OR 1.47 [95% CI 1.30–1.66, *P* < .001]) (*Figure 2*). Among the nine studies^5,6,10,12,17,20,21,23,25^ (*n* = 13 953) reporting multivariate adjusted estimates available for extraction, this excess risk was attenuated but persisted (OR 1.21 [95% CI 1.01–1.45, *P* = .038]) (*Figure 3*).

**Figure 2:**
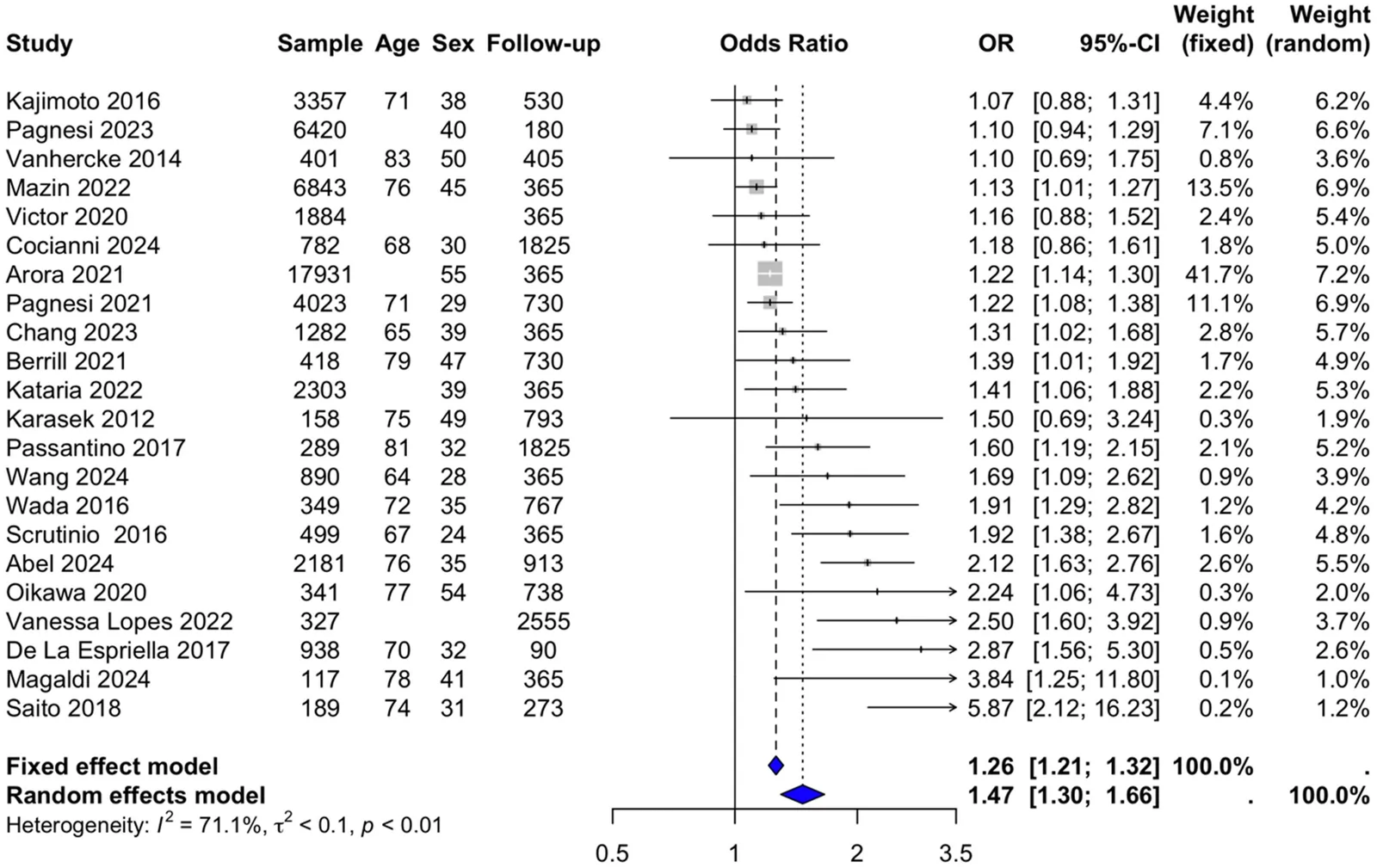
The impact of significant mitral regurgitation on all-cause mortality among all studies reporting unadjusted comparisons

**Figure 3:**
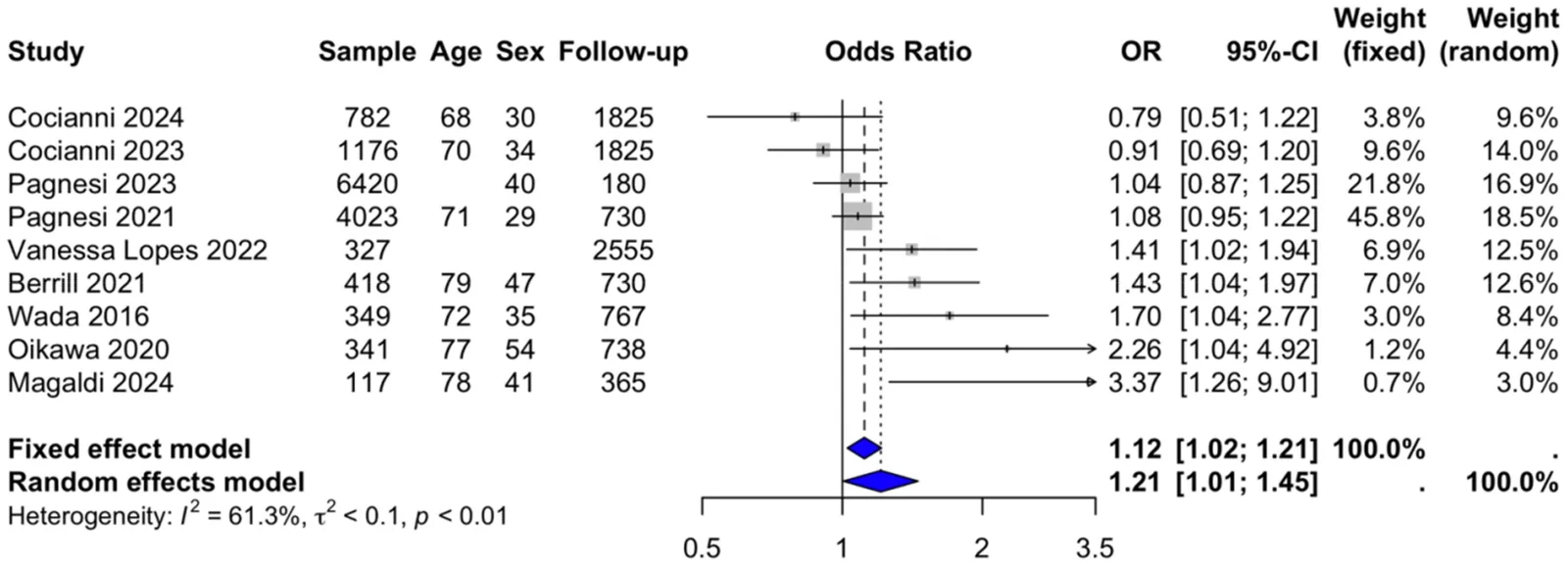
The impact of mitral regurgitation on all-cause mortality among studies reporting adjusted analyses of the primary outcome

### Secondary analyses

In a secondary analysis, compared with no MR, studies which compared moderate^12,14,16,19,26,27^ and severe MR^12,14,16,19,26–28^ suggested an increasing risk of death: moderate MR (OR 1.48 [95% CI 1.1–2.01, *P* = .011]) and severe MR (OR 1.60 [95% CI 1.13–2.27, *P* = .008]). Mild MR^3,14,19,26,27^ displayed a numerically higher but non-significant risk (OR 1.22 [95% CI .98–1.53, *P* = .079]) (*Figure 4*).

**Figure 4:**
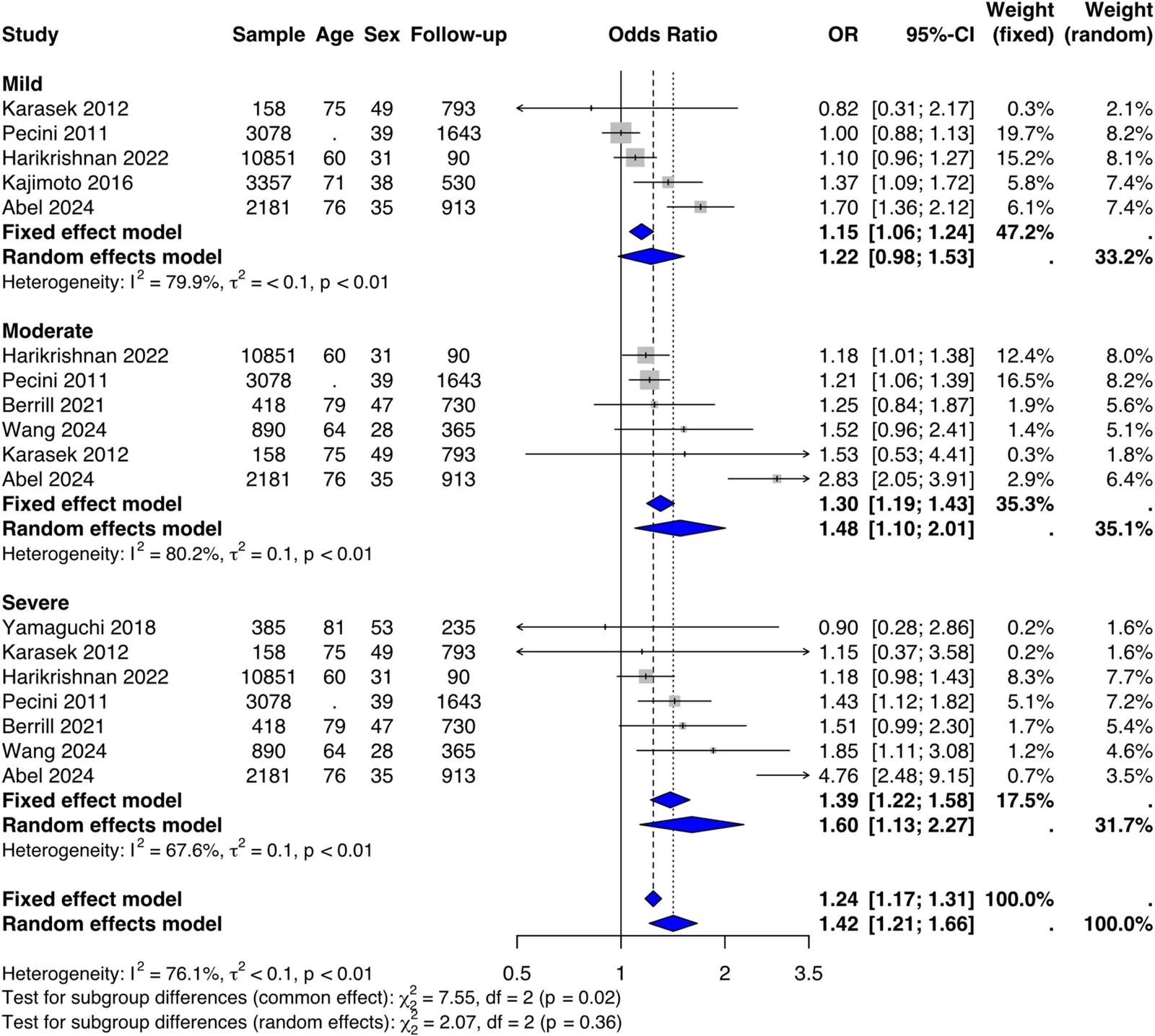
The association between mitral regurgitation severity and all-cause mortality

Among eight studies^3,4,8,9,22,24,28^ delineating MR by an LVEF <50%, the presence of moderate/severe MR was associated with an increased unadjusted risk of mortality compared with no/mild MR (OR 1.27 [95% CI 1.18–1.35, *P* < .001]). In six studies investigating those with an LVEF >50%,^3,4,8,20,24,28^ moderate/severe MR was not associated with an increased risk of mortality compared with no/mild MR (OR 1.16 [95% CI .91–1.47, *P* = .23]) (*Figure 3*). In two studies,^16,19^ including patients with LVEF 40%–50%, moderate/severe MR was associated with a greater risk of death (OR 2.00 [95% CI 1.59–2.5, *P* < .001]) (*Figure 5*).

**Figure 5:**
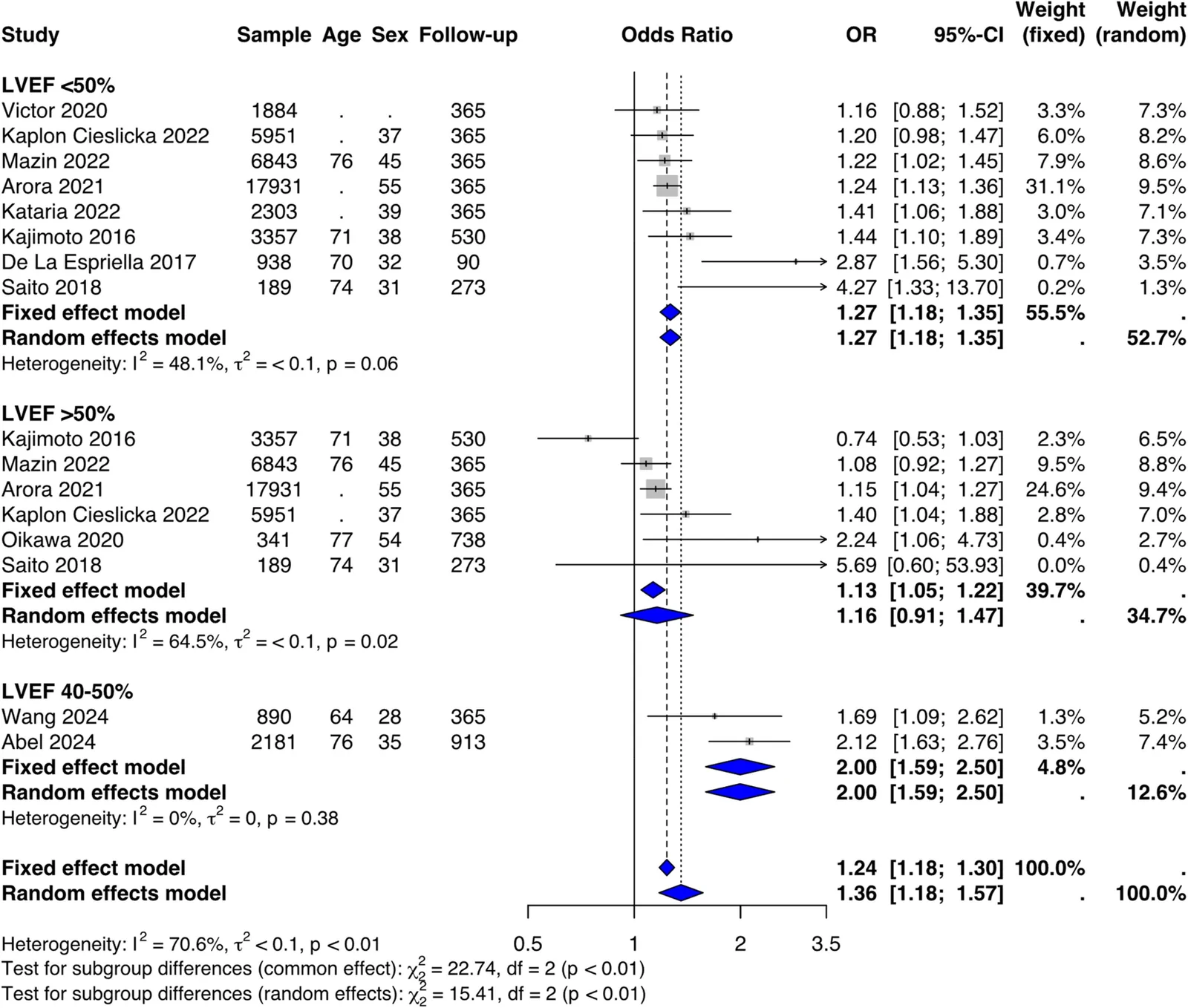
The impact of mitral regurgitation on all-cause mortality among those with left ventricular ejection fraction <50%, >50%, and 40%–50%

### Risk of bias assessment and publication bias

Five studies^9,14,17,24,27^ were considered at high risk of bias and two studies^7,29^ at medium risk (Supplementary Table S2). Removal of trials at high risk, or those at either high or moderate risk, did not change the findings of the primary analysis (Supplementary Figures S1 and S2). Publication bias was assessed using a Funnel plot (Supplementary Figure S3). There was asymmetry and the results of Egger’s test were also suggestive of publication bias.

### Meta-regression and sensitivity analysis

In two meta-regression analyses, the association between MR and outcome persisted after adjusting for age, gender, and follow-up time (OR 1.55 [95% CI 1.32–1.82, *P* < .001]) and when also adjusting for hypertension, diabetes, the presence of AF, and the LVEF (OR 1.63 [95% CI 1.28–2.06, *P* < .001]). A leave-one-out sensitivity analysis did not identify an outsized effect of any single study (Supplementary Figure S4).

## Discussion

We present a meta-analysis investigating MR in AHF and show that the presence of moderate/severe MR is associated with a 47% greater likelihood of death over a mean follow-up of nearly 2 years. This association persisted after pooling adjusted ORs and increased with the severity of MR. A 20% excess risk of death which may be attributable to MR over 2 years is clinically meaningful and may act as a target for future therapies. Key limitations, such as heterogeneity and variability in the adjustments of important confounders, limit a robust assessment of independent association; however, and the findings of this study should be considered hypothesis-generating.

An association between MR and mortality in AHF has been controversial. Large studies such as the BIOSTAT-CHF^5^ or the ESC-HFA Long-Term registry^28^ suggest an independent excess risk in moderate/severe MR, but this contrasted with the post-hoc analysis of a neutral randomized, controlled trial of serlaxin, RELAX-AHF.^6^ Meanwhile, in the large observational Atherosclerosis Risk in the Community study^4^ and a post-hoc analysis of the neutral, randomized, parallel group trial ECHOS,^26^ MR was associated with outcome among only those with lower EF. This study has attempted to assess more broadly an association with all-cause death, as well as defining MR’s impact across different LVEF phenotypes.

We suggest that MR assumes importance during AHF because of its impact on both cardiac filling pressures and forward flow. Acute changes in influx from the regurgitant volume of MR congest the pulmonary veins, causing or exaggerating dyspnoea and hypoxaemia, before loading the LV. This haemodynamic impact on a non-compliant myocardium can exacerbate ventricular filling pressures. Lack of compensatory cardiac and vascular remodelling may also lead to right ventricular-pulmonary artery uncoupling due to pulmonary venous hypertension, which can become irreversible.

The regurgitant volume is also a loss of systemic stroke volume and is wasted myocardial work. This loss reduces effective cardiac output by reducing forward flow, which may contribute to impaired perfusion. Importantly, the regurgitant volume of MR contributes to the LVEF, but not to the systemic, effective stroke volume. The use of LVEF as a surrogate of LV systolic performance is therefore problematic in the context of moderate and severe MR. However, the current paradigm of HF phenotyping (both in clinical practice and within the literature) is to delineate patients based on ejection fraction and this meta-analysis reflects this. The isolated use of the LVEF in MR and AHF, without considering filling pressures and loading conditions, we think is misleading.

We did not observe a significant risk of excess death in those with an LVEF >50%. However, we would argue that the endpoint of all-cause mortality in heart failure with preserved ejection fraction (HFpEF) is challenging to interpret. Many individuals with HFpEF have a substantial, non-cardiac comorbid burden with competing risks of death which MR might not influence. We hypothesize that MR will be associated with cardiovascular death or cardiac composite outcomes such as rehospitalization, among this cohort and further investigations into this would be clinically relevant. Further, as mentioned previously, the interaction between MR and the LVEF may also complicate studies investigating the effect of MR on outcome in this population. For example, in severe MR, an LVEF of 50%–60% is considered mildly reduced, due to loss of forward flow. An individual with an LVEF of 50% and severe MR, in AHF, may represent a phenotype of decompensated mildly reduced LVEF.

The current paradigm of MR evaluation, assessed within this study, is to determine the severity of MR in absolute terms. We continue to suggest^12^ that MR which is disproportionate to LV volumes may portend worse outcome in the acute setting, as others have originally suggested in chronic HF.^30^ We suggest this is due to a disproportionate impact on the flow and pressure changes of significant MR discussed earlier. Fifty millilitres of regurgitant MR volume, i.e. moderate MR, we hypothesize has profoundly different haemodynamic impacts within a normal sized LV of 100 ml compared with a dilated LV of 200 ml. Yet these two lesions are currently characterized solely on the absolute values of the regurgitant MR. The evaluation of disproportionate MR in the acute setting would be an interesting area of further prospective research. Future studies could compare outcomes between those with volumetrically defined MR disproportionate and those with MR proportionate to LV volumes.

The included studies were mainly observational, adjustments for important confounders such as treatment or pulmonary pressures were missing and there was a high degree of heterogeneity observed. Mitral regurgitation as an independent prognostic driver of outcome is therefore difficult to robustly assess from this meta-analysis. An alternative explanation for the observed association identified in the manuscript is that the severity of MR simply reflects a more advanced stage of HF, rather than a direct mediator of outcome per se.

This study was designed to investigate the impact of MR in the general HF patient population. We therefore excluded studies dealing with specific patient populations such as congenital heart disease, Takotsubo cardiomyopathy, or those who needed mechanical support or transcatheter interventions. Similarly, conditions in which MR is a proven driver of outcome, such as HF due to papillary muscle rupture or infective endocarditis, were also excluded. We chose all-cause death as an outcome as it is an objective, clinically meaningful endpoint which avoids misclassification bias. However, future investigations which evaluate MR’s impact on rehospitalization events or quality of life metrics would be interesting and relevant to patients.

Most studies were deemed at low risk of bias. When excluding those at high or moderate risk, this did not significantly affect the findings of the primary analysis. There was evidence of publication bias which may have enhanced any effect size. However, when we removed those at high, or moderate, risk of bias—predominantly smaller trials—and performed leave-one-out analysis, we did not observe a change in the main trial results. Publication bias may be present as we included studies which reported the impact of MR on mortality when performing multivariate adjustments as part of investigations into other variables associated with outcome in AHF. Other studies which performed this analysis and did not identify MR as affecting outcome may have been less likely to report data publicly.

### Limitations

Several limitations of our study need to be noted. Since MR is dynamic and affected by loading conditions that change with diuresis, the timing of imaging influences MR severity. There was no standardized reporting of the timing of echocardiography between studies. Individuals who have echocardiograms performed later in their admission will likely have received greater degrees of diuresis. The severity classifications used to delineate patients will therefore be affected depending on the timing of echocardiography affecting the effect estimations. We could not adequately adjust for this in our meta-analysis, likely contributing to the observed substantial heterogeneity. We did, however, include the earliest reported echocardiographic data included in each study’s results but this remains a major limitation of the study conclusions.

Furthermore, this study pools data from both functional and degenerative MR which have distinct mechanisms of pathology. Similarly, within secondary MR, our analysis did not delineate patients with atrial vs ventricular MR which again also have separate mechanisms of pathology. This may have contributed to the high degree of heterogeneity as the relative contributions of primary MR patients to varying types of secondary MR patients could have impacted the mortality rates.

As most studies were observational, we do not infer a causal relationship. To mitigate against this, we undertook multivariate analyses and meta-regression, neither of which altered our main findings. Notwithstanding our mitigation efforts, residual confounding is still possible. Definitions of AHF and methods of MR evaluation varied across studies with some relying on physician diagnoses for AHF, which may introduce bias without objective measures like natriuretic peptides. We also only included studies published in English, introducing potential language bias. Finally, two studies excluded severe MR and limited comparisons to no/mild vs moderate MR, possibly reducing the observed effect size.

The observed heterogeneity was high, although there was consistency in the direction of the measured effect. Our subgroup analyses identified likely important contributors. Different studies enrolled varying proportions of HFpEF, heart failure with mildly reduced EF (HFmrEF), and heart failure with reduced EF (HFrEF) patients, and different proportions of individuals displaying mild, moderate, and severe MR. Due to the high heterogeneity, the pooled estimates should be interpreted with caution, limiting any inference of an independent association.

### Summary

Moderate/severe MR during AHF is associated with increased mortality. Individuals with MR may represent a phenotype of AHF secondary to altered haemodynamics. Due to the observational nature of the included studies, however, residual confounding remains likely and causality cannot be inferred, and the observed heterogeneity was substantial. Further randomized trials are warranted to investigate whether therapies aimed at reducing MR can improve AHF outcome.

## Supplementary Material

xvag200_Supplementary_Data

## Declarations

### Data Availability

No data were generated or analysed for this manuscript.

### Pre-registered Clinical Trial Number

None supplied.
