# Supplementary material for: Mitral regurgitation and all-cause mortality in acute heart failure: a meta-analysis
Source: ESC Heart Fail. 2026 Aug 4;13(6):xvag200. doi: 10.1093/eschf/xvag200 (PMC13752517; doi:10.1093/eschf/xvag200)
Supplement: Supplementary file 1 [file xvag200_supplementary_data.docx]

***Table S1* – The baseline characteristics of included studies**

| **Study Author** | **Year** | **Sample Size (n)** | **Follow up (days)** | **MR assessment** | **Centred** | **Direction** |
| --- | --- | --- | --- | --- | --- | --- |
| Abel^19^ | 2024 | 2181 | 913 | Quantitative | Single-centred | Retrospective |
| Arora^4^ | 2021 | 17931 | 365 | N/a | Multi-centred | Prospective |
| Berrill^12^ | 2021 | 418 | 730 | Quantitative | Single-centred | Prospective |
| Chang^11^ | 2023 | 1282 | 365 | N/a | Single-centred | Retrospective |
| Cocianni^25^ | 2023 | 1176 | 1825 | Quantitative | Single-centred | Retrospective |
| Cocianni^10^ | 2024 | 782 | 1825 | Quantitative | Single-centred | Retrospective |
| De La Espriella^22^ | 2017 | 938 | 90 | Semi-quantitative | Single-centred | Prospective |
| Harikrishnan^27^ | 2022 | 10851 | 90 | N/a | Multi-centred | Prospective |
| Kajimoto^3^ | 2016 | 3357 | 530 | Semi-quantitative | Multi-centred | Prospective |
| Kaplon- Cieslicka^29^ | 2022 | 5951 | 365 | N/a | Multi-centred | Retrospective |
| Karasek^14^ | 2012 | 158 | 793 | N/a | Single-centred | Prospective |
| Kataria^13^ | 2022 | 2303 | 365 | N/a | Single-centred | Retrospective |
| Magaldi^23^ | 2024 | 117 | 365 | N/a | Single-centred | Prospective |
| Mazin^8^ | 2022 | 6843 | 365 | Quantitative | Single-centred | Retrospective |
| Oikawa^20^ | 2020 | 341 | 738 | Semi-quantitative | Multi-centred | Prospective |
| Pagnesi^5^ | 2021 | 4023 | 730 | N/a | Multi-centred | Retrospective |
| Pagnesi^6^ | 2023 | 6420 | 180 | N/a | Multi-centred | Retrospective |
| Passantino^15^ | 2017 | 289 | 1825 | N/a | Multi-centred | Retrospective |
| Pecini^26^ | 2011 | 3078 | 1643 | N/a | Multi-centred | Retrospective |
| Saito^24^ | 2018 | 189 | 273 | Semi-quantitative | Single-centred | Retrospective |
| Scrutinio^18^ | 2016 | 499 | 365 | N/a | Multi-centred | Retrospective |
| Vanessa Lopes^21^ | 2022 | 327 | 2555 | N/a | Single-centred | Retrospective |
| Vanhercke^7^ | 2014 | 401 | 405 | N/a | Multi-centred | Prospective |
| Victor^9^ | 2020 | 1884 | 365 | N/a | Multi-centred | Retrospective |
| Wada^17^ | 2016 | 349 | 767 | Semi-quantitative | Single-centred | Prospective |
| Wang^16^ | 2024 | 890 | 365 | Quantitative | Single-centred | Prospective |
| Yamaguchi^28^ | 2018 | 385 | 235 | N/a | Single-centred | Retrospective |

**Table S2**: Newcastle-Ottawa Scores for included studies

|  | **Selection** | | | | | **Comparability of Study Groups** | | | **Outcome of Interest** | | | | | | |  |  |
| --- | --- | --- | --- | --- | --- | --- | --- | --- | --- | --- | --- | --- | --- | --- | --- | --- | --- |
| **Author** | Representativeness | Selection of non-exposed | Ascertainment of exposure | Outcome not present | **Risk of Bias** | Primary Control | Additional Control | **Risk of Bias** | Assessment of Outcome | | Length of follow-up | | Adequacy of Follow-up | | **Risk of Bias** |  | **Overall Rating** |
| Abel | 1 | 1 | 1 | 1 | Low | 1 | 1 | Low | 1 | | 1 | | 1 | | Low |  | Low |
| Arora | 1 | 1 | 1 | 1 | Low | 1 | 1 | Low | 1 | | 1 | | 0 | | Medium |  | Low |
| Berrill | 1 | 1 | 1 | 1 | Low | 1 | 1 | Low | 1 | | 1 | | 1 | | Low |  | Low |
| Chang | 1 | 1 | 1 | 1 | Low | 0 | 1 | Medium | 1 | | 1 | | 1 | | Low |  | Low |
| Cocianni^25^ | 1 | 1 | 1 | 1 | Low | 0 | 1 | Medium | 1 | | 1 | | 1 | | Low |  | Low |
| Cocianni^10^ | 1 | 1 | 1 | 1 | Low | 1 | 1 | Low | 1 | | 1 | | 1 | | Low |  | Low |
| De La Espriella | 1 | 1 | 1 | 1 | Low | 1 | 1 | Low | 1 | | 1 | | 0 | | Medium |  | Low |
| Harikrishnan | 1 | 1 | 1 | 1 | Low | 1 | 1 | Low | 0 | | 1 | | 0 | | High |  | High |
| Kajimoto | 1 | 1 | 1 | 1 | Low | 1 | 1 | Low | 1 | | 1 | | 1 | | Low |  | Low |
| Kaplon Cieslicka | 1 | 1 | 1 | 1 | Low | 1 | 1 | Low | 0 | | 1 | | 1 | | Medium |  | Low |
| Karasek | 1 | 1 | 1 | 1 | Low | 1 | 1 | Low | 0 | | 1 | | 0 | | High |  | High |
| Kataria | 1 | 1 | 1 | 1 | Low | 1 | 1 | Low | 1 | | 1 | | 0 | | Medium |  | Low |
| Magaldi | 1 | 1 | 0 | 1 | Medium | 1 | 1 | Low | 1 | | 1 | | 1 | | Low |  | Low |
| Mazin | 1 | 1 | 1 | 1 | Low | 1 | 1 | Low | 1 | | 1 | | 0 | | Medium |  | Low |
| Oikawa | 1 | 1 | 1 | 1 | Low | 1 | 1 | Low | 1 | | 1 | | 0 | | Medium |  | Low |
| Pagnesi^5^ | 1 | 1 | 1 | 1 | Low | 1 | 1 | Low | 0 | | 1 | | 1 | | Medium |  | Low |
| Pagnesi^6^ | 1 | 1 | 1 | 1 | Low | 1 | 1 | Low | 1 | | 1 | | 1 | | Low |  | Low |
| Passantino | 0 | 1 | 1 | 1 | Medium | 1 | 1 | Low | 1 | | 1 | | 1 | | Low |  | Low |
| Pecini | 1 | 1 | 1 | 1 | Low | 1 | 1 | Low | 1 | | 1 | | 1 | | Low |  | Low |
| Saito | 0 | 1 | 1 | 1 | Medium | 1 | 1 | Low | 0 | | 1 | | 0 | | High |  | High |
| Scrutinio | 1 | 1 | 1 | 1 | Low | 1 | 1 | Low | 1 | | 1 | | 1 | | Low |  | Low |
| Vanessa Lopes | 0 | 1 | 1 | 1 | Medium | 0 | 1 | Medium | 0 | | 1 | | 0 | | High |  | High |
| Vanhercke | 0 | 1 | 1 | 1 | Medium | 1 | 1 | Low | 0 | | 1 | | 1 | | Medium |  | Medium |
| Victor | 1 | 1 | 0 | 1 | Medium | 0 | 0 | High | 0 | | 1 | | 0 | | High |  | High |
| Wada | 1 | 1 | 0 | 1 | Medium | 1 | 1 | Low | 0 | | 1 | | 0 | | High |  | High |
| Wang | 1 | 1 | 1 | 1 | Low | 1 | 1 | Low | 1 | | 1 | | 1 | | Low |  | Low |
| Yamaguchi | 1 | 1 | 1 | 1 | Low | 0 | 1 | Medium | 0 | | 1 | | 1 | | Medium |  | Medium |

***Figure S1* – Unadjusted analysis of MR’s impact on mortality - with removal of trials deemed at high risk of bias**

***Figure S2*** – **Unadjusted analysis of MR’s impact on mortality - with removal of trials deemed at high, or moderate, risk of bias**

***Figure S3* – Funnel Plot assessing publication bias**

***Figure S4:* Leave-one-out sensitivity analysis**
